# Gut‐Derived Lactic Acid Bacteria, *Pediococcus pentosaceus*, Enhances Growth Performance and Resistance to Pathogenic *Vibrio harveyi* of Hatchery‐Bred Milkfish (*Chanos chanos*) in Nursery Culture

**DOI:** 10.1155/ijm/6489487

**Published:** 2026-04-30

**Authors:** Ludevito S. Batilong, Rex Ferdinand M. Traifalgar, Carmelo S. del Castillo, Kate Alyssa G. Jore, Issa Ricci L. Fantonalgo, Fredson H. Huervana, Therese F. Javellana, Mary Jessa Bell B. Pagapulan, Alan N. Failaman, Vyenge Erre D. Gayosa

**Affiliations:** ^1^ Institute of Aquaculture, College of Fisheries and Ocean Sciences, University of the Philippines Visayas, Miagao, Iloilo, Philippines; ^2^ College of Agriculture and Fisheries, Capiz State University-Pontevedra Campus, Pontevedra, Capiz, Philippines; ^3^ Graduate School of Engineering, The University of Osaka, Osaka, Japan, osaka-u.ac.jp

**Keywords:** lactic acid bacteria, milkfish, *Pediococcus*, probiotic, *Vibrio*

## Abstract

Hatchery‐bred milkfish (*Chanos chanos*) fry continue to face stocking challenges due to inferior growth performance and reduced resilience compared with wild‐caught fry. Recent metagenomic studies have shown that wild fry harbor a higher relative abundance of bacterial taxa belonging to the phylum Bacillota. These include lactic acid bacteria (LAB), which are widely recognized for their probiotic potential in aquaculture. Building on this metagenomic insight, the present study adopted a targeted approach to isolate LAB with anti‐*Vibrio* activity and evaluate its probiotic potential in hatchery‐bred *C. chanos* nursery culture. *Screening identified Pediococcus pentosaceus* HLAB22 *as a promising LAB candidate, which was subsequently assessed through in vivo probiotic trials.* Probiotic supplementation significantly improved growth performance, survival, and reduced the incidence of opercular deformities in early juveniles. The most pronounced effects observed at 10^6^ CFU, followed by 10^3^ CFU g^-1^ feed compared with the control group. Gut colonization experiment demonstrated that dietary supplementation with *P. pentosaceus* HLAB22 at 10^6^ CFU g^-1^ feed enabled intestinal colonization within 12 days. This also resulted in near‐complete suppression of *Vibrio* populations, supporting the significant decrease in water and *C. chanos* gut *Vibrio* load during the feeding trial. Furthermore, during immersion challenge with pathogenic *V. harveyi*, the in vitro anti‐*Vibrio* activity of *P. pentosaceus* HLAB22 was translated into enhanced *in vivo* protection, yielding a survival rate of 83.33% in treated fish compared with 33.33% in the control group. Collectively, these findings indicate that oral application of *P. pentosaceus* HLAB22 at 10^6^ CFU g^-1^ feed is an effective strategy for promoting growth and enhancing resilience in *C. chanos* nursery culture. This study supports the use of targeted, host‐associated probiotics to improve the performance of hatchery‐bred milkfish fry and mitigate key challenges in nursery production systems.

## 1. Introduction

Probiotics have been extensively investigated in aquaculture as functional dietary supplements that can modulate the gut microbiome and improve host health, growth efficiency, and disease resistance [1–3]. Through the selective enrichment of beneficial microorganisms, probiotic supplementation contributes to the stabilization of intestinal microbial communities, inhibits the growth of pathogenic bacteria, and enhances host physiological performance. These effects are particularly pronounced during early developmental stages, when the gut microbiome is still undergoing assembly and is highly responsive to dietary and environmental modulation.

Milkfish (*Chanos chanos*) represents one of the most economically important finfish aquaculture species in the Philippines [4], contributing significantly to national fish production and food security. Despite sustained industry expansion over recent decades [5], milkfish production has experienced intermittent stagnation [6]. This trend has been attributed primarily to constraints in fry availability, heavy dependence on hatchery‐produced seed, and persistent challenges during nursery rearing [7, 8].

Early developmental stages, particularly within the critical window of approximately 21–51 days post‐hatch (dph), are characterized by heightened physiological stress and incomplete immune development, rendering fry highly susceptible to environmental fluctuations and infectious diseases [9–11]. As a result, hatchery‐bred fry often exhibit inferior growth, survival, and resilience during nursery rearing, leading many farmers to prefer wild‐caught fry, which are widely regarded as more resilient and better adapted to variable culture conditions [7, 12].

Among the major biological constraints, disease outbreaks, particularly vibriosis caused by luminous *Vibrio harveyi*, remain a significant challenge during the larval and nursery stages of milkfish culture [13, 14]. These recurrent disease events continue to compromise hatchery performance and underscore the urgent need for alternative health management strategies that reduce reliance on chemotherapeutics and antibiotics.

Recent advances in gut metagenomics have provided new insights into the microbial factors that potentially underlie differences in performance between wild‐caught and hatchery‐bred *C. chanos* fry [12]. Comparative analyses revealed that although both groups are dominated by bacterial families such as Roseobacteraceae and Vibrionaceae, wild fry harbor a higher relative abundance of bacteria belonging to the phylum Bacillota. Functional inference further indicated marked divergence in metabolic, cellular, organismal, and environmental response pathways between the two microbiomes. These findings suggest that the gut microbiome of wild fry may possess greater functional versatility and resilience, enabling better growth and survival adaptation to variable and potentially harsher environmental conditions. Such microbial attributes represent a promising target for translational application in hatchery and nursery systems.

Lactic acid bacteria (LAB), taxonomically classified within the phylum Bacillota, class Bacilli, order Lactobacillales, and family Lactobacillaceae, are among the most intensively studied probiotic groups in aquaculture [1, 2]. LAB are widely recognized for their ability to modulate intestinal microbial balance, suppress pathogenic bacteria through the production of organic acids and bacteriocins, enhance immune responsiveness, and improve nutrient utilization and growth performance [15–17]. While LAB‐based probiotics have been successfully applied across a broad range of cultured finfish and crustacean species, their targeted use in *C. chanos* aquaculture, particularly during the larval and nursery stages, remains poorly explored.

Based on these considerations, the present study aimed to isolate LAB from wild‐caught milkfish fry and evaluate their application as probiotics for hatchery‐bred fry. By leveraging host‐associated LAB derived from wild conspecifics, this study adopts a microbiome‐informed strategy to improve growth performance, disease resistance, and survival of milkfish during nursery culture.

## 2. Methodology

### 2.1. Ethical Statement

This study was conducted in compliance with the Philippine Republic Act No. 8485 (Animal Welfare Act of 1998) and Department of Agriculture Administrative Order No. 40, which regulates the ethical conduct of scientific research involving animals. All experimental procedures followed the standards and protocols established by the University of the Philippines Visayas Institutional Animal Care and Use Committee (UPV IACUC) to ensure the humane care and use of research animals. In addition, the study protocol was reviewed and approved by the Ethics Committee of the Institute of Aquaculture, College of Fisheries and Ocean Sciences, University of the Philippines Visayas (CFOS‐IA).

### 2.2. Isolation and Identification of Anti‐*Vibrio* LAB

Wild‐caught *C. chanos* fry (8.17 ± 1.82 mg mean body weight; 1.70 ± 0.26 cm mean total length) were collected from the coastal waters of Hamtic, Antique, Philippines and transported live to the laboratory of the Institute of Aquaculture, College of Fisheries and Ocean Sciences. A total of 50 fry was used for bacterial isolation. Under aseptic conditions, whole gastrointestinal tracts were excised and homogenized in sterile natural saline solution (0.85% NaCl). Serial 10‐fold dilutions were prepared up to 10^−7^, and aliquots (100 *μ*L) from appropriate dilutions were spread‐plated onto de Man, Rogosa, and Sharpe (MRS) agar. Plates were incubated at 30°C for 24–48 h. Following incubation, 30 morphologically distinct colonies were randomly selected to represent phenotypic diversity. Each isolate was repeatedly sub‐cultured on fresh MRS agar until pure cultures were obtained and maintained for subsequent screening.

**Figure 1:**
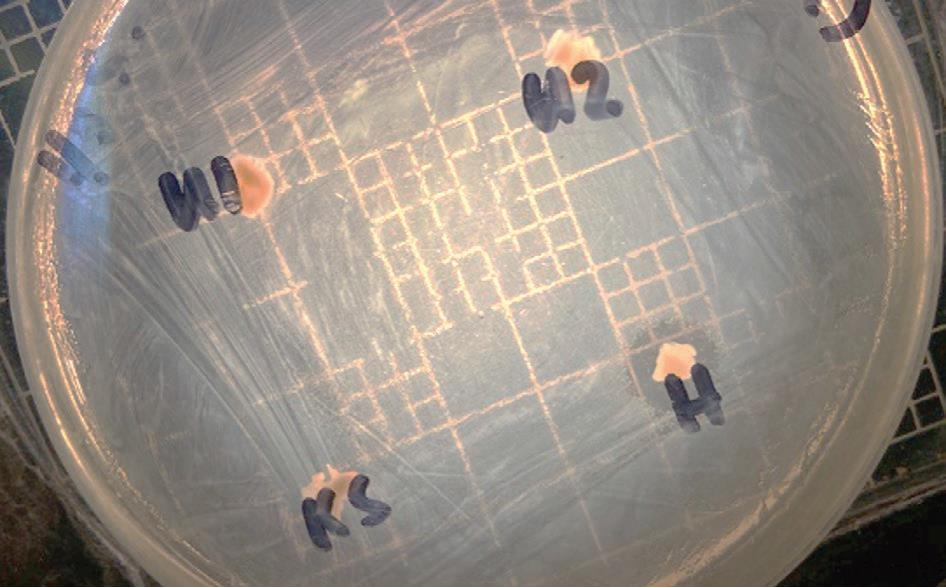
Anti‐*Vibrio* activity of *Pediococcus pentosaceus* (H) against the pathogenic *Vibrio harveyi*. Other isolates (U1, U2, and KS) showed no antagonistic activity.

Purified isolates were screened for antagonistic activity against *Vibrio harveyi* using a spot‐on‐lawn assay following Barcenal et al. [18] and Temario et al. [19] with minor modifications. Briefly, *V. harveyi* was cultured to approximately 10^7^ CFU mL^−1^ and spread uniformly onto one‐third‐strength Luria–Bertani agar supplemented with 1.5% NaCl to prepare bacterial lawns. Twenty‐four‐hour‐old LAB cultures were individually spotted onto the prepared lawns and incubated at 30°C for 24 h. Antagonistic activity was determined by the presence and measurement of inhibition zones surrounding the spotted colonies. Among the 30 isolates tested, one isolate consistently exhibited the strongest inhibitory activity against *V. harveyi* (Figure 1) and was selected for molecular identification.

**Figure 2:**
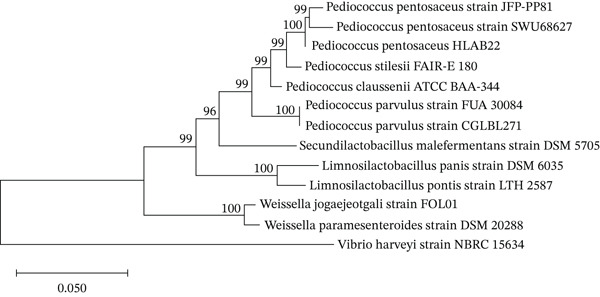
Neighbor‐joining phylogenetic tree showing the relationship of *Pediococcus pentosaceus* HLAB22 and other closely related strains based on partial 16S rRNA gene sequences. The tree was constructed using the Kimura 2‐parameter [20] model with 1000 bootstrap replications in MEGA version 12. *Lactobacillus plantarum* was used as the outgroup.

Molecular identification of the selected isolate was performed based on 16S rRNA gene sequencing. Genomic DNA was extracted using the Wizard Plus SV Minipreps DNA Purification System (Promega Corporation) following the manufacturer’s protocol. The 16S rRNA gene was amplified by polymerase chain reaction (PCR) using a T10 Thermal Cycler (Bio‐Rad Laboratories, Inc.) with universal primers 27F (5 ^′^‐AGAGTTTGATCCTGGCTCAG) and 1492R (5 ^′^‐TACCTTGTTACGACTT). Sequencing of the amplified PCR products was performed by Macrogen (South Korea). The resulting sequence was compared with reference sequences in the GenBank database using the BLAST algorithm of the National Center for Biotechnology Information (NCBI). Based on molecular characterization, the isolate showed high sequence similarity to *Pediococcus pentosaceus* and was designated *Pediococcus pentosaceus* HLAB22 (Figure 2). The 16S rRNA gene sequence was deposited in GenBank under accession number PX876037.

### 2.3. Milkfish Fry Collection and Management

Twenty‐eight‐day post‐hatch (dph) milkfish (*C. chanos*) fry were sourced from the Aquaculture Department of the Southeast Asian Fisheries Center, Tigbauan, Iloilo, Philippines. Fish were transported to the multi‐species hatchery complex of the Institute of Aquaculture, College of Fisheries and Ocean Sciences, University of the Philippines Visayas, where they were acclimatized to a 3‐ton fiberglass tank for 3 days. Commercial milkfish diets were fed ad libitum throughout the acclimation period.

### 2.4. Safety Assessment of the Probiotic Candidate

The pathogenicity of *P. pentosaceus* HLAB22 was evaluated in *C. chanos* fry following the method of Albances & Traifalgar [21], with modifications. Milkfish fry (mean body weight: 14.14 ± 0.16 mg) were randomly stocked in 12 60‐L tanks containing 56 L of seawater (34–35 ppt) at a density of 25 fish per tank. The fry were assigned to four treatments: a control group and three probiotic‐supplemented groups receiving *P. pentosaceus* HLAB22 at 10^3^, 10^6^, and 10^9^ CFU g^−1^ feed, with each treatment conducted in triplicate. The bacterial suspension was prepared by harvesting a 24‐h culture of *P. pentosaceus* HLAB22, resuspending it in sterile NSS, and serially diluting it to achieve the target concentrations. The bacterial suspensions were then incorporated into the commercial feed by spraying and thoroughly mixing 150 *μ*L of suspension per gram of feed. The control diet was prepared similarly using NSS without bacterial inoculation. Fish were fed the experimental diets twice daily (at 08:00 and 16:00 h) at 30% of their body weight. The feeding trial lasted 15 days. Throughout the experimental period, fish were monitored daily for any adverse clinical signs and mortality to evaluate the pathogenicity of the probiotic candidate.

### 2.5. Probiotic Application and Growth Evaluation

To evaluate the effects of *P. pentosaceus* HLAB22 on *C. chanos* growth performance, survival, and opercular deformities, a completely randomized design with three treatments was employed in triplicate. The experimental diets included a control diet without probiotic supplementation (Control), a diet supplemented with *P. pentosaceus* HLAB22 at 10^3^ CFU g^−1^ feed (10^3^ CFU), and a diet supplemented with 10^6^ CFU g^−1^ feed (10^6^ CFU). Probiotic suspensions were prepared by serial dilution from a stock culture standardized to 0.5 McFarland turbidity, corresponding to 10^8^ CFU mL^−1^. A total of 225 *C. chanos* fry (mean body weight: 2.54 ± 0.00 mg; mean body length: 0.992 ± 0.03 cm) were randomly distributed into 12 tanks (56 L working volume, 34–35 ppt salinity) at a density of 25 fry per tank. The fish were fed the experimental diets five times daily (08:00, 10:00, 12:00, 14:00, and 16:00 h) at a daily feeding rate equivalent to 30% of body weight. The growth evaluation experiment was run for 30 days. Biomass sampling was conducted every 10 days to adjust feed rations and monitor mortalities and deformities. Meanwhile, microbial analysis was performed every 7 days. Gut and water samples from each treatment were serially diluted (1:10), and dilutions ranging from 10^−2^ to 10^−5^ were spread‐plated on thiosulfate–citrate–bile salts–sucrose (TCBS) agar supplemented with 2% NaCl. Total *Vibrio* colonies were enumerated after 24 h of incubation at 30°C. The antagonistic effect of the probiotic was assessed by comparing *Vibrio* counts in gut and water samples among treatments. During the trial, water quality was kept within optimal levels, with temperature ranging from 27°C to 28°C, pH at 7.9–8.2, dissolved oxygen above 5 mg L^–1^, salinity between 34 and 35 ppt, ammonia from 0 to 0.15 mg L^–1^, and nitrite from 0 to 0.25 mg L^–1^. At the end of the feeding trial, the fish in each tank were harvested and bulk‐weighed to determine growth performance indices, including percent weight gain (WG), total length gain, feed conversion efficiency (FCE), and survival rate, following the formulas of De Leon et al. [22]. The incidence of opercular deformities was recorded and calculated using the following formula:

Opercular deformities %=No.of fish with opercular deformityTotal number of fish∗100

### 2.6. Gut Colonization Trial

Based on the results of the growth trial, the treatment showing the best performance (10^6^ CFU g^−1^ feed) was further evaluated for its gut colonization ability in *C. chanos* early juveniles (0.11 ± 0.00 g mean weight). Two treatments were evaluated in triplicate: a control group without probiotic supplementation and a group supplemented with *P. pentosaceus* HLAB22 at 10^6^ CFU g^–1^ feed. Fish were randomly stocked in each tank at 30 fish per tank. The bacterial suspension was prepared and incorporated into the feed following the same procedure used in the feeding trial. Fish were fed twice daily (08:00 and 16:00 h) at a feeding rate equivalent to 30% of body weight. Microbial sampling was conducted every 3 days. Nine fish from each treatment (three per replicate tank) were collected, and the intestines were aseptically excised. Fish from each treatment were collected, and the intestines were aseptically excised. Serial dilutions up to 10^−5^ were prepared, and dilutions from 10^−2^ to 10^−5^ were spread‐plated. Gut *Vibrio* spp. were enumerated on TCBS agar, while LAB were enumerated on MRS agar using the spread plate method. The presence of the test probiotic in the gut was assessed using colony replica plating on a lawn of *V. harveyi*. Colonies displaying both a zone of inhibition and the characteristic morphology of *P. pentosaceus* HLAB22 were counted to quantify gut colonization [21]. Feeding and sampling continued until complete colonization of the probiotic in the gut was confirmed.

### 2.7. *V. harveyi* Challenge Test

To evaluate the effect of *P. pentosaceus* HLAB22 probiotic on the disease resistance of nursery *C. chanos*, a *V. harveyi* immersion challenge test was conducted following the methods of Gayosa et al. [10]. Ninety early juveniles, averaging 0.16 ± 0.13 g (mean ± SEM) and previously fed either a control diet or a diet supplemented with *P. pentosaceus* HLAB22 at 10^6^ CFU/g feed, were used. Fish were randomly assigned to three groups, each with three replicate tanks containing 10 fish per tank. The negative control group was maintained in UV‐filtered seawater without bacterial exposure and received the control diet. The positive control group was fed the control diet and exposed to a *V. harveyi* suspension (10^5^ CFU mL^−1^ calculated using Reed‐Muench for LD50). The probiotic‐treated group received the probiotic‐supplemented diet and was exposed to the same bacterial suspension. Immersion was performed in a static bath for 1 h in 2 L of ozonated seawater (34–35 ppt) with moderate aeration (DO: 5–6 mg L^−1^), with no water exchange or feeding throughout the experiment to maintain a consistent bacterial load. Fish were monitored three times daily (08:00, 12:00, and 16:00 h) for behavior, lesion development, and mortality, with unresponsive individuals considered dead if they did not react to the touch of a glass rod. At the end of the experiment, three moribund fish exhibiting fin rot or skin lesions from each group, along with control fish, were randomly selected for bacterial re‐isolation from the gut using Nutrient agar with 1.5% NaCl and TCBS to confirm *V. harveyi* infection. Cumulative survival was calculated using the formula used by [23].

### 2.8. Statistical Analysis

Statistical analyses were performed using SPSS v.29. One‐way analysis of variance (ANOVA) was applied to assess growth parameters and Vibrio loads in culture water and the gut of *C. chanos*, followed by Tukey’s HSD test for pairwise comparisons at *p* < 0.05. Data from the gut colonization assay, including *Vibrio* and LAB counts, as well as survival against *V. harveyi*, were analyzed using Student’s *t*‐test at the same confidence level. All data are presented as mean ± SEM.

## 3. Results

### 3.1. Pathogenicity of *P. pentosaceus* HLAB22

The pathogenicity trial of *P. pentosaceus* HLAB22 in *C. chanos* showed that feeding fry with up to 10^9^ CFU g^−1^ feed for 15 days did not induce any clinical signs of disease or mortality. These results indicate that the isolate is non‐pathogenic and safe for use as a probiotic in *C. chanos* nursery operations.

### 3.2. Growth Performance Effects

Dietary supplementation with *P. pentosaceus* HLAB22 significantly improved the growth performance of *C. chanos* fry (Table 1). Percent WG was highest in fish fed diets containing 10^6^ CFU g^−1^ feed, followed by those receiving 10^3^ CFU, and was lowest in the control group. A similar pattern was observed for other growth indices, including specific growth rate (SGR) and FCE. Survival rates were also significantly enhanced in *Pediococcus*‐treated groups, with the highest survival recorded in fish fed 10^6^ CFU. In addition, the incidence of opercular deformity decreased significantly with increasing dietary *P. pentosaceus* HLAB22 concentration.

**Table 1:** Growth performance metrics of *Chanos chanos* nursery applied with *Pediococcus pentosaceus* HLAB22 at different concentrations for 30 days.

Treatment	FBW	WG	SGR	FCE	OPD	Survival
Control	121.35 ± 4.99	4677.48 ± 195.75^c^	12.88 ± 0.14^c^	51.97 ± 2.17^c^	67.50 ± 9.53^a^	33.00 ± 3.46^c^
10^3^ CFU	148.65 ± 4.99	5752.29 ± 195.41^b^	13.56 ± 0.11^b^	63.91 ± 2.17^b^	19.00 ± 3.46^b^	44.00 ± 4.04^b^
10^6^ CFU	168.65 ± 4.99	6539.69 ± 195.26^a^	13.98 ± 0.10^a^	72.66 ± 2.17^a^	12.00 ± 6.35^b^	82.00 ± 8.08^a^
*p* value	0.002	0.002	0.002	0.002	0.002	0.002

*Note:* Values are shown as mean ± SEM. Initial body weight = 2.54 ± 0.00 mg. Survival (%). Values are presented as mean ± SEM (*n* = 3). Within columns, different superscripts indicate significance (*p* < 0.05).

Abbreviations: FBW = final body weight (mg); FCE = feed conversion efficiency; OPD = opercular deformity (%); SGR = specific growth rate (% day^−1^); WG = weight gain (%).

### 3.3. *Vibrio* Load Associated With *C. chanos* Nursery Culture

**Figure 3:**
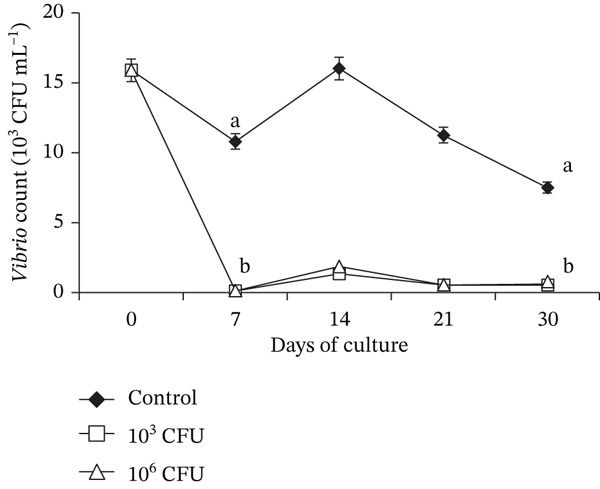
Presumptive *Vibrio* levels in the culture water of *Chanos chanos* applied with varying dietary *Pediococcus pentosaceus* probiotic concentrations. Values are shown as mean ± SEM. Different superscripts indicate significance (one‐way ANOVA with Tukey’s HSD, 0 DOC: *p* = 1.000; 7 DOC: *p* ≤ 0.001; 14 DOC: *p* ≤ 0.001; 21 DOC: *p* ≤ 0.001; 30 DOC: *p* ≤ 0.001).

**Figure 4:**
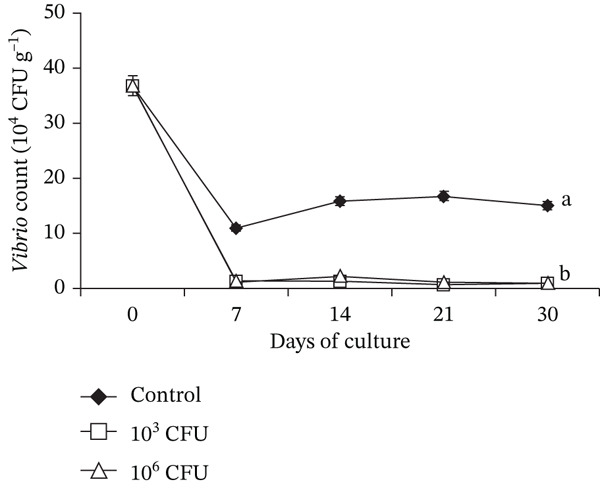
Presumptive *Vibrio* abundance in the gut of early juvenile *Chanos chanos* fed diets containing different concentrations of *Pediococcus pentosaceus* probiotic. Values are shown as mean ± SEM. Significance is denoted by difference in superscripts (one‐way ANOVA with Tukey’s HSD, 0 DOC: *p* = 1.000; 7 DOC: *p* ≤ 0.001; 14 DOC: *p* ≤ 0.001; 21 DOC: *p* ≤ 0.001; 30 DOC: *p* ≤ 0.001).

*Vibrio* counts in the culture water of *C. chanos* were significantly reduced following dietary supplementation with *P. pentosaceus* HLAB22 (Figure 3). Diets containing 10^3^ and 10^6^ CFU g^−1^ feed produced comparable reductions in intestinal *Vibrio* levels (Figure 4). In contrast, control tanks exhibited stable to increasing *Vibrio* counts in both environments throughout the experimental period. Overall, data shows that *P. pentosaceus* HLAB22, even at a low concentration of 10^3^ CFU g^−1^, can inhibit total *Vibrio* in both water and *C. chanos* gut within 7 days.

### 3.4. Gut Colonization Efficiency of *P. pentosaceus* HLAB22

**Figure 5:**
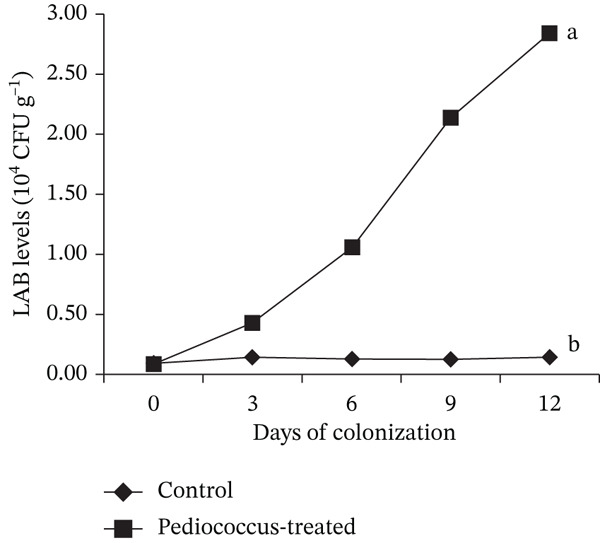
Gut colonization of MRS‐culturable LAB with phenotypic characteristics consistent with *P. pentosaceus* HLAB22. Values are shown as mean ± SEM. Statistical significance is indicated by different superscripts (Student’s *t*‐test, 0 DOC: *p* = 0.678; 3 DOC: *p* ≤ 0.001; 6 DOC: *p* ≤ 0.001; 9 DOC: *p* = 0.002; 12 DOC: *p* ≤ 0.001).

**Figure 6:**
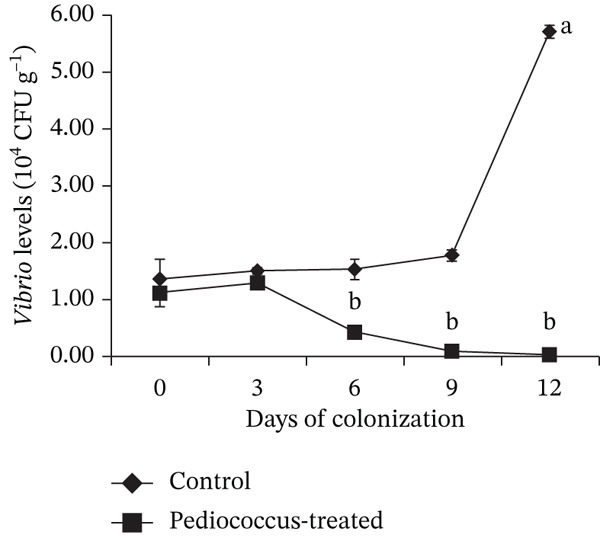
Changes in presumptive *Vibrio* abundance in the gut of *Chanos chanos* during colonization by *Pediococcus pentosaceus*. Values are shown as mean ± SEM. Different superscripts indicate significance (Student’s *t*‐test, 0 DOC: *p* = 0.852; 3 DOC: *p* = 0.012; 6 DOC: *p* = 0.004; 9 DOC: *p* ≤ 0.001; 12 DOC: *p* = 0.007).

Results showed that feeding *C. chanos* early juveniles with a diet supplemented with *P. pentosaceus* HLAB22 at 10^6^ CFU g^−1^ feed for 12 days resulted in successful gut colonization (Figure 5) and a marked reduction in intestinal *Vibrio* populations (Figure 6). In the control group, *Vibrio* levels increased significantly from Day 0 to Day 12, rising from 1.36 × 10^4^ to 5.71 × 10^4^ CFU g^−1^ tissue. In contrast, fish receiving *P. pentosaceus* HLAB22 exhibited a significant reduction in *Vibrio* abundance over the same period, decreasing from 1.11 × 10^4^ to 2.00 × 10^2^ CFU g^−1^ tissue, indicating near‐complete suppression of *Vibrio* pressure in the gut. Based on these outcomes, the trial was terminated at Day 12, as this feeding duration was sufficient to demonstrate probiotic establishment and its antagonistic effect against *Vibrio*.

### 3.5. Milkfish Early Juveniles’ Resistance to Pathogenic *V. harveyi*

**Figure 7:**
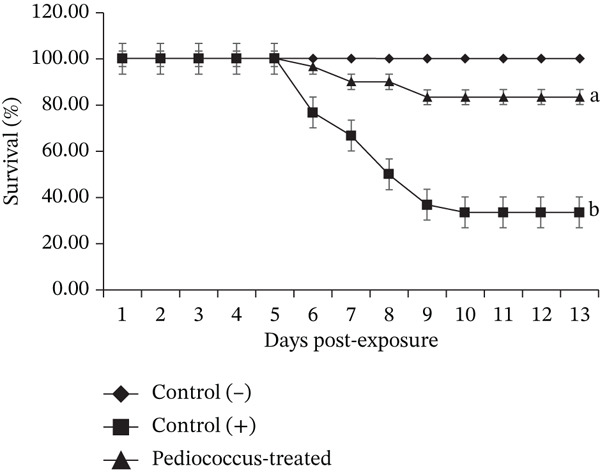
Survival of *Chanos chanos* early juveniles fed control and *Pediococcus pentosaceus*‐supplemented diets at 10^6^ CFU g^−1^ feed following challenge with pathogenic *Vibrio harveyi*. Values are shown as mean ± SEM. Different superscripts indicate significant differences (Student’s *t*‐test, 6 DPE: *p* = 0.004; 10 DPE: *p* = 0.047).

Dietary supplementation with *P. pentosaceus* HLAB22 at 10^6^ CFU g^−1^ feed significantly improved the survival of *C. chanos* early juveniles following challenge with pathogenic *V. harveyi* (Figure 7). Fish fed the control diet exhibited the lowest resistance, with a survival rate of 33.33%. In contrast, fish receiving *P. pentosaceus* HLAB22 showed markedly enhanced resistance, achieving a survival rate of 83.33%, representing a 50‐percentage‐point increase compared with the control group. No mortality was observed in the negative control group, indicating that the observed effects are directly attributable to the probiotic treatment.

## 4. Discussion

LAB are among the most widely studied probiotic microorganisms in aquaculture due to their ability to modulate gut microbiota, enhance nutrient utilization, and stimulate host immune responses [24–28]. Members of this group have demonstrated beneficial effects on growth performance, disease resistance, and overall health of cultured aquatic organisms. Among LAB, *Pediococcus* spp., members of the family Lactobacillaceae, have received increasing attention because of their antimicrobial property and probiotic potential. Positive correlations between growth performance and *Pediococcus* supplementation have been reported in various aquaculture species, including fish and crustacean [15, 17]. However, their application to enhance *C. chanos* nursery performance remains largely unexplored. To our knowledge, this study represents the first report of *Pediococcus pentosaceus* applied as a probiotic in *C. chanos* nursery culture.

The results of the feeding trial and growth evaluation demonstrated that supplementation with *P. pentosaceus* HLAB22 at best dose of 10^6^ CFU g^−1^ feed led to significant improvements in WG, SGR, FCE, and survival, as well as a reduction in opercular deformities in *C. chanos* fry. Comparable growth‐promoting effects of *P. pentosaceus* have been reported in other aquaculture species, including Pacific whiteleg shrimp (*Litopenaeus vannamei*) [29], mud crab (*Scylla paramamosain*) [30], common carp (*Cyprinus carpio*) (E. [31]), grass carp (*Ctenopharyngodon idella*) [32], rainbow trout (*Oncorhynchus mykiss*) [33], and red sea bream (*Pagrus major*) [34].

Other *Pediococcus* species have also been shown to enhance growth performance in zebrafish (*Danio rerio*) (M. [35]), European sea bass (*Dicentrarchus labrax* L.) (E.‐S. H. [36]), rainbow trout (*O. mykiss*) [37], and Persian sturgeon (*Acipenser persicus*) [38]. Notably, these studies employed higher probiotic doses (10^7^–10^12^ CFU), whereas in the present study, lower concentrations (10^3^–10^6^ CFU g^−1^ feed) were sufficient to elicit beneficial effects in early juvenile *C. chanos*. These findings support the potential of *Pediococcus* spp. as effective growth‐promoting probiotics in fish culture.

The growth‐promoting effects of *Pediococcus* on *C. chanos* observed in the present study could be attributed to the reduction of *Vibrio* populations in both the rearing water and the gut of *C. chanos*. This effect may be associated with the production of antimicrobial compounds by *Pediococcus* spp., such as penocin and pediocins, which exhibit broad‐spectrum antibacterial activity against numerous fish pathogens [16]. Consistent with these findings, previous studies have reported that *Pediococcus* spp. exert potent bactericidal effects against pathogenic *Vibrio* species, including *V. anguillarum*, *V. parahaemolyticus*, and *V. alginolyticus*, as well as other pathogens such as *Aeromonas hydrophila*, *A. veronii*, *A. sobria*, *Edwardsiella tarda*, *Lactococcus garvieae*, *Plesiomonas shigelloides*, *Staphylococcus aureus*, and *β*‐*Streptococcus* and are associated with enhanced growth performance and immunomodulatory responses in grass carp (*C. idella*), Pacific whiteleg shrimp (*L. vannamei*), and mud crab (*S. paramamosain*) [29, 30, 32], similar to the outcomes observed in the present study. Although the present work did not identify the specific antimicrobial compound produced by the local isolate, *P. pentosaceus HLAB22* demonstrated clear antibacterial activity against *V. harveyi*, as evidenced by the spot‐on‐the‐lawn screening assay.

Further, previous studies report that *Pediococcus* spp. are capable of producing digestive enzymes, including proteases, amylases, and lipases, which enhance nutrient degradation and assimilation when applied as probiotics in aquatic hosts [39–41]. This enzymatic activity likely assists the host fish in improving nutrient breakdown and bioavailability, as observed in *D. rerio* and *C. idella*, and may also explain the growth‐promoting effects seen in the present study ([32, 35]; Mohammadi [42]).

In addition to improving growth performance, probiotic supplementation also reduced the incidence of opercular deformities in *C. chanos* fry in the present study. Opercular deformities are among the most commonly observed skeletal abnormalities in cultured fish larvae and juveniles. The high incidence of this abnormality in the control group (67.50%) is associated with nutritional deficiencies, environmental stress, and microbial infections during early developmental stages [36, 43]. Such deformities may impair proper gill coverage and respiratory efficiency, potentially affecting fish health, survival, and overall production performance in aquaculture systems.

The reduced occurrence of opercular deformities in fish receiving *P. pentosaceus* HLAB22 may be linked to the improved physiological condition resulting from probiotic‐mediated enhancement of digestive efficiency, aiding the release of vitamins and fatty acids which can support proper skeletal and tissue development during early life stages [44–46]. The successful colonization of *P. pentosaceus* HLAB22 in the gut of *C. chanos* further contributed to the reduction of total *Vibrio* loads to safe levels (< 10^4^ CFU; [13, 47]). This decrease in pathogenic pressure may have resulted in less energy being allocated to inflammatory responses due to infection, allowing more energy to be channeled into normal skeletal development, growth, and tissue synthesis [10, 48].

The inhibitory activity of *P. pentosaceus* HLAB22 against *V. harveyi* has resulted in improved survival of fish receiving 10^6^ CFU g^−1^ feed of the probiotic when challenged with the pathogenic *V. harveyi*. Consistent with our present findings, enhanced survival following *Pediococcus* application has been documented in grouper (*Epinephelus coioides*) challenged with *V. anguillarum* [49], in *Oreochromis* spp. exposed to *Aspergillus flavus* and *Aeromonas* spp. (M. E. H. [50–52]), and in mud crab (*S. paramamosain*) challenged with *V. parahaemolyticus* [30]. These results underscore the functional role of *Pediococcus* in gut microbial modulation and highlight its potential as a sustainable and effective strategy for disease management in aquaculture.

## 5. Conclusion

The present study shows that probiotic application of *P. pentosaceus* HLAB22 at a dose of 10^6^ CFU g^−1^ feed improved growth, promoted feed utilization, enhanced culture survival, reduced larval opercular deformities, as well as increased the resistance of *C. chanos* when exposed to pathogenic *V. harveyi*. The application of *P. pentosaceus* could be a practical approach to improving growth performance, lowering larval deformities, and enhancing disease resistance in hatchery‐bred *C. chanos* reared under nursery culture conditions. While the present work shows promising results, future research could explore the long‐term effects of *P. pentosaceus* HLAB22 on growth, skeletal development, and disease resistance, as well as the underlying mechanisms through gut microbiome and immune response analyses. Additionally, studies on optimal dosing and potential synergistic effects with other probiotics or functional feeds would help maximize its practical application in aquaculture.

## Funding

This study was supported by the Philippine Council for Agriculture, Aquatic and Natural Resources Research and Development, (DOST‐PCAARRD) under the project Improvement of Milkfish Larval Rearing and Nursery Culture through Gut Metagenome, Transcriptome Analysis, and Gut Microbial Community Manipulations. 10.13039/501100014166.

## Conflicts of Interest

The authors declare no conflicts of interest.

## Data Availability

The data that support the findings of this study are available from the corresponding author upon reasonable request.
